# Pharmacological Alterations of Anxious Behaviour in Mice Depending on Both Strain and the Behavioural Situation

**DOI:** 10.1371/journal.pone.0007745

**Published:** 2009-11-11

**Authors:** Yan Clément, Anne-Marie Le Guisquet, Patrice Venault, Georges Chapouthier, Catherine Belzung

**Affiliations:** 1 Université Reims Champagne-Ardenne, Reims, France; 2 Psychobiologie des émotions, Université François Rabelais de Tours, Faculté des Sciences et Techniques, Parc Grandmont, Tours, France; 3 Université Pierre et Marie Curie-Paris 6, CNRS, UMR 7225, Inserm, U 975, Paris, France; 4 USR CNRS 3246 Groupe Hospitalier Pitié-Salpêtrière, Paris, France; University of Queensland, Australia

## Abstract

A previous study comparing non-emotive mice from the strain C57BL/6/ByJ with ABP/Le mice showed ABP/Le to be more anxious in an open-field situation. In the present study, several compounds affecting anxiety were assayed on ABP/Le and C57BL/6/ByJ mice using three behavioural models of anxiety: the elevated plus-maze, the light-dark discrimination test and the free exploratory paradigm. The compounds used were the full benzodiazepine receptor agonist, chlordiazepoxide, and the antagonist, flumazenil, the GABA_A_ antagonist, bicuculline, the full 5-HT_1A_ agonist 8-OH-DPAT, and the mixed 5-HT_1A_/5-HT_1B_ agonist, RU 24969. Results showed the effect of the compounds to be dependent on both the strain and the behavioural task. Several compounds found to be anxiolytic in ABP/Le mice had an anxiogenic effect on C57BL/6/ByJ mice. More behavioural changes were observed for ABP/Le in the elevated plus-maze, but the clearest findings for C57BL/6/ByJ mice were observed in the light-dark discrimination apparatus. These data demonstrate that anxious behaviour is a complex phenomenon which cannot be described by a single behavioural task nor by the action of a single compound.

## Introduction

Anxiety is a widespread phenomenon occurring in response to various stressors. In humans, there is not one single syndrome, but several which may explain different anxiety conditions reported and could provide evidence for hypotheses on the involvement of certain biological substrates [1], [2]. Anxiety in animals is less clear, given the obvious difficulty in assessing psychological components, but it has been suggested that anxiety is not a single phenomenon. Studies of rodents have assessed anxiety using animal models of fear, e.g. the light/dark test and the elevated plus-maze paradigm to measure state anxiety, and the free exploratory test to measure trait anxiety [3]–[10].

It has been suggested that several factors, environmental and genetic, can be seen in the aetiology of anxiety, with genetic factors in both humans and animals regulating the physiological processes involved in anxiety [11], [12]. Studies have shown phenotypic differences in inbred strains of mice [13]–[19] and many *loci* have been associated with an increase in the behavioural expression of anxiety [11], [20]–[22]. When the genetic factors involved are located on eight or more chromosomes, the behaviour patterns are said to depend on a multigenic system and the genetic background [1], [23]. The strain ABP/Le (hereafter ABP) strain was found to be more anxious than C57BL/6/ByJ (hereafter B6) and the 4^th^, 7^th^ and 9^th^ murine chromosomes were found to be associated with anxiety [13], [14]. Since two of the three chromosomes (7^th^ and 9^th^ chromosomes) putatively involved in anxiogenic processes contain loci encoding for either the GABA_A_ receptor subunits (α5, β3, γ3) or for the 5-HT_1B_ receptor, the hypothesis of a biochemical correlate with anxiogenic behaviour patterns was tested. Binding studies were conducted and the anxious phenotype was found to be present with modifications caused by the BZ antagonist [^3^H]flumazenil and the 5-HT_1B_ receptor agonist [I^125^]cyanolopindolol [24], [25].

Many previous studies have found clear evidence of the anxiolytic effects of GABA_A_-BZ receptor ligands [26]–[28]. Benzodiazepine (BZ) agonists have anxiolytic properties, whereas BZ inverse agonists have an anxiogenic effect. Studies of *in vivo* administration of serotoninergic (5-HT) ligands have failed to find clear evidence of either an anxiolytic or an anxiogenic effect when administered to animals, including effects dependent on the behavioural model, the dose or the 5-HT receptor subtype.

Full 5-HT_1A_ agonists have, however, often been seen to produce anxiolytic effects in animals, yet in humans partial agonists are used to relieve anxiety [29]–[32]. Activation of the 5-HT_1B_ receptor can also increase anxiety [33], [34]. A number of pharmacological studies have been confirmed by experimental studies using knockout animals. Decreased GABA_A_-receptor clustering, and inactivation of the gene coding for the 5-HT_1A_ receptor have been seen to induce anxiety-like behaviour [35]–[39]. Observations of 5-HT_1B_ receptor knockout mice, however, did not find any consistent modification in anxiety levels [40], [41].

The present study set out to investigate the hypothesis that the GABA_A_-BZ and 5-HT neurotransmission systems may be involved in an animal model of anxiety, and that genetic factors may determine differential sensitivity to specific drugs. In line with previous studies [4], [10], [42], the behavioural analysis was conducted after *in vivo* administration of one of a number of compounds: the full BZ agonist, chlordiazepoxide (5 mg/kg), the BZ antagonist, flumazenil (3 mg/kg), the GABA_A_ antagonist, bicuculline (1 mg/kg), the 5-HT_1A_ agonist 8-OH-DPAT (0.3 mg/kg) and the mixed 5-HT_1A_/5-HT_1B_ agonist, RU 24969 (2.5 mg/kg). Three animal models of anxiety were used: the elevated plus-maze, the light-dark test procedure and the free exploratory paradigm. The study was designed as a pilot study to analyse two different transmitter systems (GABA-BZ and 5-HT). For ethical reasons to minimise the number of animals used, only one dose of each compound was administered. The compounds were selected for their relevance as reported in the literature.

## Materials and Methods

### Animals

The animals were male and female mice bred in the laboratory from two parent strains: ABP/Le (n = 151) and C57BL/6ByJ (n = 185). They were reared under standard conditions: temperature 23.5±0.5°C. A 12:12 h photoperiod with lights on at 8:00 am, tap water and Souriffarat (IM UAR) feed available *ad libitum*, and dust-free softwood sawdust bedding. Litters were culled to 7 subjects at birth. From birth to weaning, the animals were kept with their mothers only; the sires were removed from the mating cages one or two days before parturition. Male and female offspring were separated when weaned at 30+2 days. The animals were 10 weeks old ± 2 weeks when tested. All experiments complied with the ethical guidelines laid down by the French Ministry of Agriculture and with the European Council Directive 86/609/EEC.

### Behavioural testing

The experiments were conducted in a room outside the breeding room between 13.00 h and 17.00 h. Data were recorded using a hand-held computer (Psion Organiser). An independent group of mice was used for each behavioural test. The tables give details of the number of animals in each. Mice were naive to the test apparatus.

### Light-dark apparatus

Two polyvinyl chloride boxes (20×20×14 cm) covered with Plexiglas were connected via a semi-opaque plastic tunnel (5×7×10 cm). One box was dark, while the other box was lit by a 100 W desk lamp 20 cm above the box; this was the only light in the room. The subjects were individually tested in 5-min sessions. The mouse was placed in the lit box to start the test session. The parameters recorded were the time spent in the lit box, the number of transitions (i.e. the mouse crossing and placing all four paws in the opposite box) and the number of entries into the tunnel after the first entry (an entry being defined as more than 2 seconds spent in the tunnel).

### Elevated plus-maze

The apparatus was a polyvinyl chloride plus-maze with two lit open arms (27×5 cm) and two closed arms (27×5×15 cm) covered with cardboard to block out the light; all four arms radiated from a central platform (5×5 cm). The apparatus was mounted on a base raising the arms to a height of 38.5 cm above the floor. To initiate the test session, the mouse was placed on the central platform, facing an open arm, and was observed for 5 min. The mouse was considered to be on the central platform whenever two paws were on it, and in one of the arms when all four paws were inside. The following behavioural variables were recorded, counting both number and duration: entries into an open arm, entry into a closed arm and unprotected head-dipping (the animal extending its head into the open, below the open arm).

### Free exploratory paradigm (Hughes Box)

The apparatus consisted of a polyvinyl chloride box (30×20×20 cm) covered with Plexiglas and subdivided into six identical square exploration units, all interconnected by small doors. A temporary partition divided the apparatus in half lengthwise. Approximately 24 h before testing, each subject was placed in one half of the apparatus, with the temporary partition in place, to be familiarised with it. The floor was covered with sawdust and the animal was given unlimited access to food and water. The next day, the same mouse was exposed to both the familiar and novel environments after the temporary partition was removed, but without the animal being removed from the box. The subject was then observed under red light for 10 min. Parameters recorded were the number of episodes of avoidance behaviour in response to the novel environment (attempts), the number of units entered (locomotion), the number of rearings and the time spent in the novel side.

### Drugs

Chlordiazepoxide HCl (Sigma, L'Isle D'Abeau, France) (5 mg/kg), suspended in a vehicle, was administered 30 min before testing. RU 24969 (Tocris, Illkirch, France) (2.5 mg/kg), dissolved in a vehicle, was administered 40 minutes before testing. 8-OH-DPAT (Sigma, L'Isle D'Abeau, France) (0.3 mg/kg) and flumazenil (donated by Hoffmann-La Roche, Basle, Switzerland) (3 mg/kg) were dissolved in saline and injected 20 minutes before testing. Bicuculline (Sigma, St. Louis, MO) (1 mg/kg) was dissolved in hot saline and mixed with acetic acid to produce a final concentration of approximately 0.01 M; this was done because bicuculline is unstable in physiological pH. The cooled solution was injected 10 minutes before testing. All the drugs were administered by intraperitoneal injection; the volume injected was 10 ml/kg body weight. The vehicle injection was saline with one drop of Tween 80 and one drop of acetic acid, and was injected 20 minutes before testing (i.e. the approximate harmonic mean of the intervals before behavioural testing). Each animal was given one injection, either an active drug or saline solution.

### Statistical analysis

A multivariate analysis of variance was performed with “Strain”, “Treatment” and “Gender” as the main components, plus their interactions using a GLM SAS procedure followed by planned contrast comparisons. Partial comparisons were done using the adjusted means with the Least Squares Means (LSMeans) statement of GLM (SAS). Strain * Treatment interaction was also tested.

## Results

### Light-dark apparatus (Table 1)

A Strain effect was observed, but only for the number of entries into the tunnel, F_1,98_ = 8.09, p = 0.005; it also showed a Treatment effect which was significant for time spent in the lit box and the number of transitions, F_5,98_ = 3.29, p = 0.009; F = 4.45, p = 0.001, respectively.

**Table 1:** Comparison (mean±S.E.M.) of ABP and B6 mice + drug treatment (mg/kg) in light-dark apparatus.

	Saline solution		8-OH-DPAT 0.3 mg		Chlordiazepoxide 5 mg		Bicuculline 1 mg	
Behaviour	ABP n = 9	B6 n = 8	ABP n = 5	B6 n = 10	ABP n = 7	B6 n = 10	ABP n = 6	B6 n = 9
Time spent in lit box	49.3±7.0	70.1±10.*	47.7±7.6	46.4±9.9	57.8±12.0	53.1±13.7 ↓	29.2±6.8	22.9±8.9 ↓
Number of transitions	6.0±0.9	7.3±1.3	5.8±0.8	5.1±0.1	5.2.7±0.7 ↑	8.1±0.8 *	4.1±0.8	2.1±1.1 ↓
Number of entries in tunnel	16.7±1.6	16.6±2.2	15.3±1.8	15.0±2.4	14.7±1.6 ↑	22.6±1.8 ↑*	12.5±1.7 ↓	7.0±2.3 ↓

	Saline solution		Flumazenil 3 mg		RU 24969 2.5 mg	
Behaviour	ABP n = 9	B6 n = 8	ABP n = 8	B6 n = 9	ABP n = 8	B6 n = 9
Time spent in lit box	49.3±7.0	70.1±10.0 *	33.7 6.2 ↓	41.2±7.8 ↓	49.8±8.0	35.3±10.5 ↓
Number of transitions	6.0±0.9	7.3±1.3	4.8±0.7	4.6±0.9	10.7±1.3 ↑	4.3±1.7 *
Number of entries in tunnel	16.7±1.6	16.6±2.2	15.5±1.8	12.5±2.3	30.8±2.1 ↑	11.4±2.7 *

NB: Figures on time spent in tunnel and time spent in dark box not given; data not significant.

*Strain difference (ABP vs. B6).

↑increased behaviour by treatment (drug mg/kg) vs. saline).

↓decreased behaviour by treatment.

The Strain * Treatment interaction was significant for the number of transitions, F_5,98_ = 4.51, p = 0.0001. Neither the Strain * Treatment * Gender interaction, nor the Strain * Gender or Treatment * Gender interactions were significant. As no Gender effect was observed, male and female data were pooled for the partial comparisons.

Partial comparisons after:

- *Saline solution* and *8-OH-DPAT* treatment: no treatment or strain effect was observed.

- *Chlordiazepoxide (CDZ)*

The number of transitions and the number of entries into the tunnel were lower for ABP than for B6, t = 6.97, p = 0.017, t = 10.51, p = 0.005, respectively.

CDZ increased the number of entries into the tunnel for B6 but not for ABP, t = 1.97, p = 0.05.

- *Bicuculline*

There was no difference in behaviour between ABP and B6. Bicuculline treatment when compared to saline did, however, reduce the time spent in the lit box and the number of transitions by B6 but not by ABP: t = 3.03, p = 0.003; t = 2.94, p = 0.006, respectively. The treatment (bicuculline vs saline) reduced the number of entries into the tunnel by both strains: t = 3.63, p = 0.007; t = 2.80,p = 0.006, respectively.

- *Flumazenil*

No strain effect on behaviour was observed between ABP and B6, but a treatment effect (flumazenil vs saline) was observed, with both B6 and ABP spending less time in the lit box: t = 1.92, p = 0.05; t = 1.76, p = 0.08, respectively.

- *RU 24969*

The number of transitions and the number of entries into the tunnel were higher for ABP than for B6: t = 2.96, p = 0.009; t = 5.60, p = 0.0003, respectively.

Compared to saline treatment, RU 24969 treatment caused a decrease in time spent in the lit box but only by B6, not by ABP, while the number of transitions and the number of entries into the tunnel increased in ABP, but not in B6: t = 2.30, p = 0.02; t = 3.66, p = 0.004, respectively.

### Elevated plus-maze (Table 2)

A Strain effect was observed for the time spent in the open arms: F _1.79_ = 71.98, p = 0.0001.

**Table 2:** Comparison (mean±S.E.M.) of ABP and B6 mice + drug treatment (mg/kg) in elevated plus-maze.

		Saline solution		8-OH-DPAT 0.3 mg		Chlordiazepoxide 5 mg		Bicuculline 1 mg	
Behaviour		ABP n = 7	B6 n = 10	ABP n = 5	B6 n = 10	ABP n = 7	B6 n = 10	ABP n = 5	B6 n = 9
Entries on the open arm	Nb.	19.6±5.2	5.0±3.5 *	13.6±5.2	3.7±2.4 *	25.4±4.4	6.6±3.7 *	25.8±5.2	3.9±1.7 *
	Dn.	41.1±17.5	14.0±1.7	62.7±17.4	10.4±12.3	85.7±14.7 ↑	16.9±12.3 *	111.4±17.5 ↑	6.8±13.0 *
Entries on the enclosed arm	Nb.	29.6±4.4	22.3±2.9	17.2±4.4 ↓	22.5±3.1	23.9±3.7	25.5±3.1	24.6±4.4	29.3±3.3
	Dn.	111.0±29.4	91.5±19.8	52.7±29.4	88.7±20.7	123.8±24.8	104.0±20.7	70.2±29.3	97.9±±21.9
Head dipping	Nb.	23.4±4.2	7.6±3.0 *	12.8±4.2 ↓	5.6±3.0	27.0±3.6	9.2±3.0 *	26.0±4.3	6.8±3.1 *
	Dn.	20.7±4.6	9.7±3.1 *	10.8±4.6	4.8±3.3	24.3±3.9	11.9±3.2 *	24.1±4.6	6.7±3.4 *

		Saline solution		Flumazenil 3 mg		RU 24969 2.5 mg	
Behaviour		ABP n = 7	B6 n = 10	ABP n = 5	B6 n = 8	ABP n = 6	B6 n = 10
Entries on the open arm	Nb.	19.6±5.2	5.0±3.5 *	32.4±5.2	2.6±4.1	42.7±4.8 ↑	9.6±3.7 *
	Dn.	41.1±17.5	14.0±1.7	91.9±17.4 ↑	5.3±13.8 *	103.2±15.9 ↑	35.6±12.3 *
Entries on the enclosed arm	Nb.	29.6±4.4	22.3±2.9	30.2±4.4	27.0±3.5	33.7±4.0	16.9±3.1
	Dn.	111.0±29.4	91.5±19.8	69.8±29.4	90.6±23.2	84.4±26.8	86.0±20.7
Head dipping	Nb.	23.4±4.2	7.6±3.0 *	28.2±4.3	5.6±3.3 *	39.7±3.8 ↑	7.3±3.0 *
	Dn.	20.7±4.6	9.7±3.1 *	23.1±4.6	5.0±3.6 *	32.3±4.2 ↑	7.8±3.2 *

Nb.  = Number; Dn.  = Duration (secondes).

*Strain difference (ABP vs B6).

↑increased behaviour by treatment (drug mg/kg vs. saline).

↓decreased behaviour by treatment.

The Treatment factor was significant for the duration head-dipping, F = 3.27, p = 0.010. A Strain * Treatment interaction was observed for head-dipping, F = 2.66, p = 0.028, respectively.

Partial comparisons after:

- *Saline treatment*

The number of entries into the open arms and the number of head-dippings were higher in ABP than in B6: t = 2.39, p 0.019; t = 1.98, p = 0.050 respectively.

- *8-OH-DPAT*

The number of entries into the open arms was higher in ABP strain than in B6, t = 2.47, p = 0.016.

The treatment caused a decrease in head-dippings and entries in the closed arms in ABP but not in B6: t = 1.75, p = 0.08; t = 2.00, p = 0.05, respectively.

- *Chlordiazepoxide*

The ABP mice spent longer and recorded more entries into the open arms as well as more head-dippings than B6: t = 3.57, p = 0.0006; t = 3.31, p = 0.002; t = 2.43, p = 0.017, respectively.

CDZ caused an increase in the time spent in the open arms by ABP, but not by B6: t = 1.95, p = 0.05.

- *Bicuculline*

The duration and number of entries into the open arms and the duration and number of head dippings were higher in ABP than in B6: t = 3.67, p = 0.0005; t = 4.80, p = 0.0001; t = 3.59, p = 0.0006, t = 3.01, p = 0.004, respectively.

Bicuculline caused an increase in the time spent in the open arms by ABP but not by B6, t = 2.84, p = 0.006.

- *Flumazenil*

The time spent in the open arms, and the number and duration of head-dippings were higher in ABP than in B6: t = 3.89, p = 0.0002; t = 4.15, p = 0.0001; t = 2.10, p = 0.039, respectively.

Flumazenil caused an increase in the time spent in the open arms by ABP but not by B6: t = 2.05, p = 0.043.

- *RU 24969*

The time spent and the number of entries into the open arms were higher in ABP than in B6, t = 3.34, p = 0.001; t = 4.45, p = 0.000, respectively. The number and duration of head-dippings were also higher in ABP strain than in B6; t = 4.59, p = 0.0001; t = 2.05, p = 0.042, respectively.

RU 24969 caused an increase in the number of entries and duration in the open arms and the number and duration of head-dippings, but only in ABP mice: t = 2.62, p = 0.01; t = 2.75, p = 0.005; t = 2.81, p = 0.006; t = 1.86, p = 0.067, respectively.

### Free Exploratory Paradigm, Hughes Box (table 3)

Three-way ANOVA showed Treatment x Strain interactions for rearing, F_5,123_ = 5.70, p = 0.001, and for time spent in the novel area, F_5,123_ = 5.46, p = 0.001. A Strain x Gender interaction was found to be significant for rearing, F_1,123_ = 4.66, p = 0.03. This appears to be the only Gender interaction with another factor, and was only small in magnitude; the interaction was therefore considered fortuitous and the factor was not included in the analysis. The Treatment factor was significant for locomotion, rearing and time spent in the novel area: F_5,123_ = 13.53, p = 0.001; F = 3.56, p = 0.005; F = 2.93, p = 0.01, respectively. A Strain effect was observed for the same parameters: F_1,123_ = 25.58, p = 0.001; F = 8.59, p = 0.004; F = 14.61, p = 0.001, respectively. The Gender effect reached significance for rearing and the time in the novel area: F_1,123_ = 7.26, p = 0.008; F = 5.84, p = 0.01, respectively. The number of attempts was not different.

**Table 3:** Comparison (mean±S.E.M.) of ABP and B6 mice + drug treatment (mg/kg)in free exploratory paradigm.

	Saline solution		8-OH-DPAT 0.3 mg		Chlordiazepoxide 5 mg		Bicuculline 1 mg	
Behaviour	ABP n = 13	B6 n = 12	ABP n = 11	B6 n = 10	ABP n = 12	B6 n = 13	ABP n = 10	B6 n = 10
Time in novel side (sec.)	481.3±11.4	410.5±11.96 *	441.2±32.5	438.6±19.4	374.5±54.4	375.0±19.4	476.3±27.1	307.2±52. ↓ *
Attempts	2.7±0.6	1.7±0.9	2.4±1.0	1.2±0.5	2.6±0.7	4.6±1.5	2.2±0.8	2.2±0.7
Locomotion	99.4±11.0	132.4±14.6	75.4±11.9	127.7±11.8 *	90.9±17.5	131.7±12.7	77.8±5.3	101.8±14.8
Rearing	33.2±5.0	40.2±5.6	17.4±3.3	33.2±3.7 *	17.3±5.5	31.5±4.3 *	27.4±4.5	34.9±6.1

	Saline solution		Flumazenil 3 mg		RU 24969 2.5 mg	
Behaviour	ABP n = 13	B6 n = 12	ABP n = 13	B6 n = 14	ABP n = 14	B6 n = 13
Time in novel side (sec.)	481.3±11.4	410.5±11.9 *	397.4±34.	417.5±11.2	461.7±20.1	290.0±28 ↓ *
Attempts	2.7±0.6	1.7±0.9	3.8±1.1	3.6±1.5	7.78±2.2	2.4±1.5 *
Locomotion	99.4±11.0	132.4±14.6	85.6±12.8	127.8 ±10.0 *	162.1±11.1 ↑	207.7±20.1 ↑ *
Rearing	33.2±5.0	40.2±5.6	19.6±4.6	44.5±4.5 *	47.8±4.6	29.2±5.1 *

*Strain difference (ABP vs B6).

↑increased behaviour by treatment (drug mg/kg vs saline).

↓decreased behaviour by treatment.

### Effects of Treatments

- *Saline treatment*

Novelty preference (time in novel side) was lower in B6 than in ABP: t = 4.28, p = 0.001.

- *8-OH-DPAT*

Locomotion and rearing were lower for ABP than for B6: t = 3.11, p = 0.005; t = 3.18, p = 0.005, respectively.

No effect of 8-OH-DPAT was seen in either strain.

- *Chlordiazepoxide*

Rearing decreased in ABP mice compared to B6: t = 2.04, p = 0.05.

No treatment effect was detected.

- *Bicuculline*

The two strains recorded different times spent in the novel side, with B6 mice displaying a lower novelty preference: t = 2.85, p = 0.01.

Bicuculline, compared with controls, caused a decrease in novelty preference, but only in B6: t = 2.08, p = 0.05.

- *Flumazenil*

The two strains had different results: locomotion and rearing were lower in ABP than in B6: t = 2.61, p = 0.01; t = 3.85, p = 0.001, respectively.

The treatment effect was not significant.

- *RU 24969*

Between-strain differences were observed for all variables tested: locomotion was lower, t = 2.02, p = 0.05; time in the novel side, attempts and rearing were higher in ABP than in B6, t = 5.03, p = 0.001; t = 2.02, p = 0.05; t = 2.73, p = 0.01, respectively.

RU 24969 increased locomotion in both ABP and B6: t = 4.00, p<0.001; t = 2.98; p = 0.007, respectively. Drug treatment decreased the time spent in the novel side in B6, but not in ABP: t = 3.84, p = 0.001.

## Discussion

An animal is usually considered anxious if it spends less time in the lit box of a light-dark apparatus, does less exploration of the open arms of a plus-maze apparatus and spends less time in the novel side of the free exploratory apparatus [4], [8], [15], [36], [43]. Using these three tests, recognised as models of anxiety in rodents, (tables 1, 2 & 3), and a comparative design with and without pharmacological treatment, we studied the anxiety behaviour of two inbred strains, ABP and B6. Saline treated controls showed strain-dependent differences in behaviour, most significantly in the plus-maze model and the free exploratory test. In the plus-maze, ABP mice recorded more entries into the open arms and more head-dippings (table 2), more rearing and grooming (data not included). In the free exploratory test, ABP mice spent more time in the novel side, suggesting they are less anxious (table 3). The behaviour cannot be linked to any difference in the level of locomotion, as not only were there no strain-related differences for entries into the closed arms, but in the free exploratory test, the general activity of B6 mice was higher than ABP mice.

It must be noted that these results do not appear to tally with previous findings obtained in open-field testing, where ABP mice were found to be more active than B6 [25]. Differences in experimental situations may account for this discrepancy and clear out a complex interaction between genetic and environmental factors [44], [45].

The pharmacological action of selected compounds was reported using the same 3 tests and 2 strains, showing, for example, that the effects on ABP mice in the elevated plus-maze were always anxiolytic, and that the effects on B6 mice in the light/dark apparatus were always anxiogenic.

Two comments can be made at this point. First, it could be argued that the ABP strain may be a better murine model for studying the anxiolytic effects of drugs, while the B6 strain would be better suited to uncovering anxiogenic effects. But some compounds were also seen to have anxiolytic effects on B6 mice [46]–[49]. Secondly, it could be argued that when experimenting with mice, the light/dark choice test may be more relevant for detecting anxiogenic effects, while the plus-maze may be better suited to measuring anxiolytic effects, even though anxiolytic effects have been clearly observed using the same version of the light/dark apparatus ([42], [50]–[52] 52 Griebel et al., 1992), and anxiogenic actions have been observed in the elevated plus-maze [53]–[56].

The administration of chlordiazepoxide (benzodiazepine receptor agonist) induced anxiolytic effects in ABP mice tested in the elevated plus-maze, confirming the anti-anxiety effects of benzodiazepine agonists which have been extensively reported. However, the same effect was not observed in ABP mice in the light/dark test, providing further evidence for the argument that the two tests assess different behaviour patterns. This is not a new discovery; diazepam has been shown to produce anxiolytic effects in Swiss, BALB/c and C3HeOuJIco mice in either the elevated plus-maze test or the light/dark choice test; yet the same compound, at the same dose, produced anxiolytic effects in C57BL/6JIco, DBA/2JIco, NMRI and NZB/Ola/Hsd mice in the elevated plus-maze, but not in the light/dark choice test [47], [57].

Flumazenil (benzodiazepine receptor antagonist) is usually described as devoid of intrinsic action in rodent models of anxiety, such as conditioned conflict paradigms [58], [59], the elevated plus-maze [60], [61], the light/dark choice test [62], the staircase [62], [63], the burying test [64] and ultrasonic vocalisations [65]. Yet flumazenil has also been described as an anxiogenic agent for testing in the elevated plus-maze [66], [67], the social interaction model [68], [69] and the mouse defence test battery [58], [70]; it has been shown to produce an inverse agonist-like promnesic effect in a learning task [71]. In some cases it has even been described as agonistic [72], [73]; e.g. rats trained to discriminate clorazepate from saline extend the cue to include flumazenil [74]. In some situations, flumazenil has been seen to induce agonist or inverse agonist-like effects, depending on the level of threat or stress [75]–[77], and on the strain used [47]. In the present study, the benzodiazepine antagonist was anxiolytic in ABP mice in the elevated plus-maze but had an anxiogenic effect on the B6 mice in the light/dark apparatus. As pharmacological reactions were induced in both strains, it is difficult to implicate pharmacokinetic differences in any explanation of behavioural differences. The treatment*strain interaction confirms that the effects of flumazenil depend upon environmental and genetic factors.

The behavioural effects of the GABA_A_ receptor antagonist, bicuculline, are also strain-dependent. Bicuculline induced anxiety in B6 mice in both the free exploratory test (a decrease in the time spent in the novel environment) and the light/dark test, but induced an anxiolytic effect on ABP mice in the elevated plus-maze. As ABP and B6 mice were both sensitive to bicuculline, the differences observed should not be related to pharmacokinetic differences. The anxiogenic effects of the GABA_A_ receptor antagonist are not surprising given that benzodiazepines are believed to produce their anxiolytic effect by increasing GABAergic neurotransmission. Another experiment obtained similar results using very high doses (up to 8 mg/kg) [78]. But no consistent evidence of an anxiogenic profile has been found [79] and it has been suggested that when anxiogenesis is observed after bicuculline administration, it may be attributed to behavioural suppression rather than to any effect on anxiety; for example, a dose of 1 mg/kg produced behavioural suppression in Swiss mice in the free exploratory test [80]. This was not found in the present study; in the free exploratory test, bicuculline had no effect on locomotion or rearing in either strain. The anxiolytic effect of the GABA_A_ receptor antagonist on the ABP strain is surprising and may be related to dysfunction of the GABA_A_ receptor.

In a previous study [25], [^3^H]flumazenil binding in brain homogenates of ABP and B6 mice was measured after exposure to a novel situation. Scatchard analysis showed greater affinity in the BZR binding sites of the ABP strain. Kd changes may indicate that certain animals considered as “more anxious” have fast adaptive cellular mechanisms, causing an increase in BZR affinity in response to novelty-induced stress. The different behaviour patterns observed in the present report after administering flumazenil or bicuculline may be explained by differential qualitative changes in both strains (i.e. changes in the molecular stoechiometry of the GABA_A_ receptors) or by a rapid post-transcriptional regulatory mechanism, such as phosphorylation of the receptor protein. The possibility, however, that different allelic forms encoding for GABA_A_ receptors may be correlated with the pharmacological profiles observed cannot be excluded. The ABP linkage-testing strain is interesting as it contains a genetic marker (*pink-eyed dilution*, 7^th^ chromosome) close to loci encoding for the α5 and β3 GABA_A_ protein receptor [81]. Many mRNAs encoding for these proteins are found in the cortex (α5 and β3) and in the hippocampus (mainly α5). As these loci co-segregate in intercrossed F2 Mendelian populations (easily identifiable animals) and since the α5 subunit has been associated with the pharmacological effects of benzodiazepines [82], further pharmacological experiments on these populations (*p/p* F2), using specific and high affinity ligands for these receptors, could clarify the putative role of these GABA and BZ binding sites in anxiety.

Administration of 8-OH-DPAT (full 5-HT_1A_ agonist) had no effect in the light-dark apparatus and there was no strain difference in drug sensitivity, confirming previous data [24], [83]. In the plus-maze and the free exploratory paradigm we observed some minor effects which also corroborated the findings of previous studies [4], [43], [61].

RU 24969, a mixed 5-HT_1A_/5-HT_1B_ agonist, produced anxiolytic effects in ABP mice in the elevated plus-maze, while it produced anxiogenic effects in B6 mice in the elevated plus-maze and the free exploratory test. A review of the literature shows RU 24969 to have either anxiolytic or anxiogenic effects, depending on the behavioural test used; it is usually anxiogenic in the elevated plus-maze test and has been reported as being anxiolytic in a modified Vogel test and in the four plate test [84]. The interaction observed in the present study is therefore not surprising and contributes new data. The administration of RU 24969 stimulated locomotion in the free exploratory test in both strains, but RU 24969 produced opposite effects on anxiety in the two strains, suggesting that while the drug affects locomotion, it may not affect the expression of anxiety. Assuming that the effects of RU 24969 on locomotion can be linked to 5-HT_1B_ within the striatum, it may be that the two strains differ in their expression of 5-HT_1B_ in other brain areas, e.g. the limbic system. The 5-HT_1B_ receptors are mainly found in extrapyramidal neural pathways, and these are mainly presynaptic terminal autoreceptors which inhibit the release of 5-HT in the cortex and substantia nigra. The 5-HT_1B_ receptors are also heteroreceptors and modulate the release of other neurotransmitters; for example, 5-HT inhibits ACh release in the hippocampus. In the globus pallidus and the substantia nigra, GABA release is inhibited by 5-HT_1B_ activation [57], [85]. In a previous study, quantitative autoradiography [I^125^] was used to measure cyanolopindolol binding sites in different areas of the brain in ABP and B6 mice [24]. An increase was observed in the density of 5-HT_1B_ in the globus pallidus and substantia nigra of the more “anxious” and more active ABP mice, confirming the involvement of striatal 5-HT_1B_ receptors in locomotion. Unfortunately no data are available on binding sites in the limbic system of the mice. The ABP strain also has a genetic marker which is close to a locus encoding for the 5-HT_1B_ subtype. The short-ear (se) locus (9^th^ chromosome), expresses itself in an easily identifiable phenotype [86]. It is thus possible to make segregating populations homozygous for the se gene, and consequently cosegregate for the 5-HT_1B_ gene. This population can be used for measuring differential mRNA 5-HT_1B_ and/or 5-HT_1B_ protein expression in areas of the limbic system such as the hippocampus. It may then be assumed that the 5-HT_1B_ gene could be mutated in the ABP strain and correlated with a differential pharmacological pattern.

The present data challenge the conventional view that the anxiolytic effect of benzodiazepines is the same regardless of the behavioural situation, although this may still be the case for certain specific strains of mice. When applied to another mouse strain, as evidenced the present study, several compounds known to be anxiolytic, displayed a clearly anxiogenic profile. Furthermore, the anxious phenotype also depends on characteristics of the behaviour test used. Finally, more data with more than one dose are indeed necessary before concluding in anxiogenic or anxiolytic effect of such a ligand, mainly because interaction between Strain X Environment X Treatment is complex.
